# Genome-Wide Analysis of Glycoside Hydrolase Family 1 β-glucosidase Genes in *Brassica rapa* and Their Potential Role in Pollen Development

**DOI:** 10.3390/ijms20071663

**Published:** 2019-04-03

**Authors:** Xiangshu Dong, Yuan Jiang, Yoonkang Hur

**Affiliations:** 1 School of Agriculture, Yunnan University, Kunming 650091, China; jiangyuan@mail.ynu.edu.cn; 2 Department of Biological Sciences, Chungnam National University, Daejeon 34141, Korea

**Keywords:** β-glucosidase, *Brassica rapa*, BrBGLU10, pollen development, co-expression analysis

## Abstract

Glycoside hydrolase family 1 (GH1) β-glucosidases (BGLUs) are encoded by a large number of genes, and are involved in many developmental processes and stress responses in plants. Due to their importance in plant growth and development, genome-wide analyses have been conducted in model plants (*Arabidopsis* and rice) and maize, but not in *Brassica* species, which are important vegetable crops. In this study, we systematically analyzed *B. rapa* *BGLU*s (*BrBGLU*s), and demonstrated the involvement of several genes in pollen development. Sixty-four *BrBGLU*s were identified in *Brassica* databases, which were anchored onto 10 chromosomes, with 10 tandem duplications. Phylogenetic analysis revealed that 64 genes were classified into 10 subgroups, and each subgroup had relatively conserved intron/exon structures. Clustering with *Arabidopsis* BGLUs (AtBGLUs) facilitated the identification of several important subgroups for flavonoid metabolism, the production of glucosinolates, the regulation of abscisic acid (ABA) levels, and other defense-related compounds. At least six BrBGLUs might be involved in pollen development. The expression of *BrBGLU10*/*AtBGLU20*, the analysis of co-expressed genes, and the examination of knocked down *Arabidopsis* plants strongly suggests that *BrBGLU10*/*AtBGLU20* has an indispensable function in pollen development. The results that are obtained from this study may provide valuable information for the further understanding of β-glucosidase function and *Brassica* breeding, for nutraceuticals-rich *Brassica* crops.

## 1. Introduction

Glycoside hydrolases (EC 3.2.1) are classified into a group of enzymes that hydrolyze the glycosidic bonds of carbohydrates [1]. At the end of March in 2019, 161 families have been identified and classified in the CAZy (Carbohydrate-Active enZYmes) database [2,3]. Among these families, the glycoside hydrolase (GH) family 1 is recognized for its β-glycosidase activity, which largely contributes to various developmental processes and stress responses in plants [4,5]. Genome-wide analysis of GH1 β-glycosidase genes (*BGLU*s) has been conducted in three plant species: *Arabidopsis*, with 48 genes grouped into 10 subfamilies [6]; rice, with 40 genes grouped into eight subfamilies [5]; and maize, with 26 genes grouped into four subfamilies [7,8]. Recently, a comparison between the *Arabidopsis* and rice *BGLU*s with respect to sequence identity and expression revealed that these exhibited substantial tissue specificity and differential responses to various stress treatments, although these have a high degree of similarity [9]. However, no systematic analysis of *BGLU*s in *Brassica rapa*, which is an important vegetable crop, has been performed to date.

In addition to classifications based on genomic DNA organization, *Arabidopsis* BGLUs (*AtBGLU*s) could be classified in relation to their known functions, which shows that genes within the same subfamily may function in similar processes. A large number of *AtBGLU*s are involved in flavonoid metabolism: *AtBGLU1-6* for flavonol accumulation [10,11], *AtBGLU7-11* for anthocyanin glucosyltransferase [11,12], and *AtBGLU12-17* for flavonoid utilization [10,13]. Seven genes (*AtBGLU26*, *AtBGLU34-39*) function as myrosinases for chemical defense against herbivores and pathogen attacks [14,15,16]. *AtBGLU18* and *AtBGLU33* regulate ABA responses by increasing ABA levels through the hydrolysis of glucose-conjugated ABA (ABA-GE) [17,18]. Scopolin, which is specifically produced in the roots, and which plays a role in a defense against pathogen attack and abiotic stresses [19,20], is controlled by *ArBGLU21-23* [21,22]. The gene products encoded by *AtBGLU45* and *AtBGLU46* hydrolyze monolignol glucosides, thereby regulating lignin biosynthesis [23]. *AtBGLU42* is involved in the induction of systemic resistance to bacterial disease, and the release of iron-mobilizing phenolic metabolites during iron deficiency [24]. However, no gene has been reported, with respect to pollen development.

During pollen development, the tapetum secretes various components, such as lipidic precursors and lipidics onto the pollen surface, leading to the formation of sculptured exine and exine cavities by hydrolyzation and other reactions [25]. In addition to lipid components, pollen wall development requires the regulation of polysaccharide metabolism [26], suggesting a possible involvement of the hydrolysis of glycosidic bonds of carbohydrates. Glycoside hydrolase has been reported involved in the cell wall polysaccharide degradation [27] and their coding genes were downregulated in the *OsTDR* (*Tapetum Degeneration Retardation*) mutant [28] and the sterile floral buds of *B. rapa* [29], indicating a possibility that β-glucosidase may play a role in pollen development.

In this study, we systematically identified *Brassica rapa* β-glycosidase genes (*BrBGLU*s) and analyzed their expression patterns and phylogenetic relationships. In addition, in silico analyses indicated that *BrBGLU10/AtBGLU20* have conserved functions during pollen development, and knocking down *AtBGLU20* using antisense oligos in *Arabidopsis* results in the production of aborted pollen grains. Furthermore, bioinformatics and molecular analyses provide valuable information on the function of *BrBGLUs* during pollen development.

## 2. Results

### 2.1. Identification and Chromosomal Distribution of BrBGLUs

**Figure 1:**
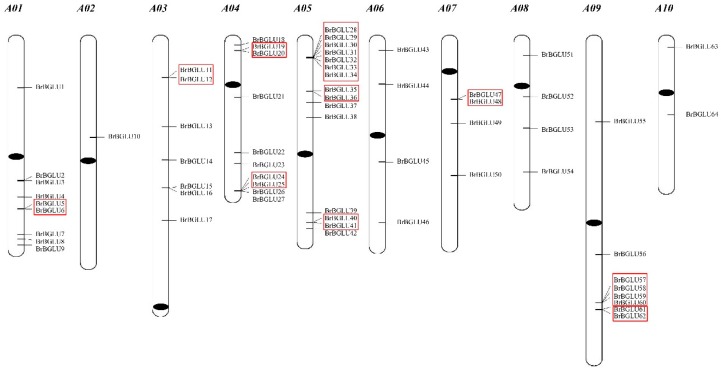
Chromosomal distribution of the 64 *BrBGLU* genes identified in this study. The chromosome number is indicated above each chromosome. Ten clusters of *BrBGLUs* are indicated in red boxes. Black ovals on each chromosome represent the centromeric regions.

After a HMM (Hidden Markov Model) search, 64 *BrBGLU* genes were identified and designated as *BrBGLU1* to *BrBGLU64*, according to their positions on the chromosomes (Figure 1). The locus ID, genome location, coding sequence (CDS) length, and the protein length of the *BrBGLUs* are listed in Table 1. The genomic DNA sequences of the *BrBGLU*s ranged from 390 bp to 9617 bp. While the average length was 1293 bp, the length of the CDS of the *BrBGLU*s ranged from 267 bp to 2058 bp. The *BrBGLU* genes were heterogeneously distributed among all 10 chromosomes of *B. rapa*. Chromosome 5 contained the largest number of *BrBGLU* genes, comprising 15 members (23.4%), whereas chromosome 2 contained only one gene member. We also detected tandem arrays of the *BrBGLU* genes among the 10 *B. rapa* chromosomes. The tandem array was defined as ‘multiple *BrBGLU* genes located in neighboring or the same intergenic region’ [30]. Ten *BrBGLU* gene clusters were found on chromosomes A01, A03, A05, A07, and A09. Chromosome 5 contained the maximum number of clusters, comprising 11 *BrBGLU*s.

### 2.2. Phylogenetic and Gene Structure Analysis of BrBGLUs

**Figure 2:**
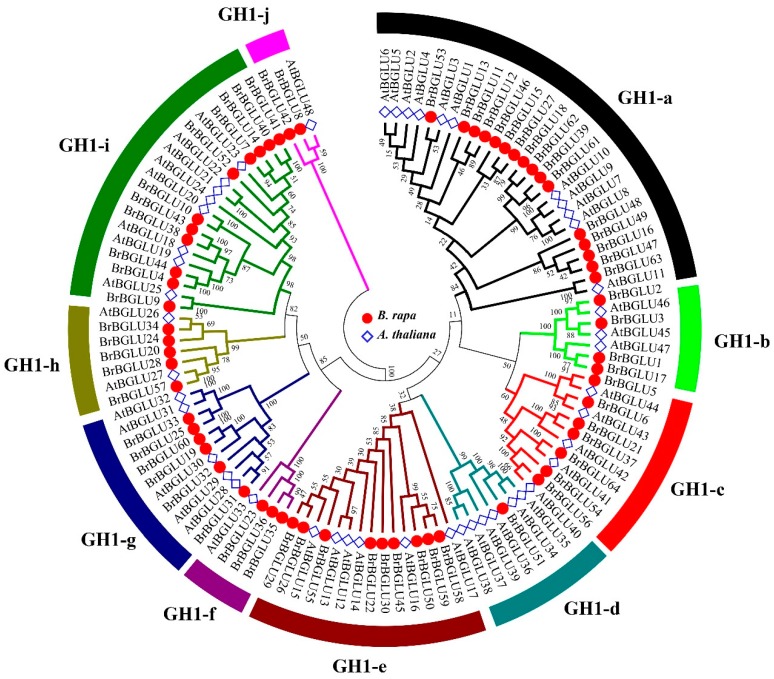
Phylogenetic reconstruction of GH1 genes of *Arabidopsis* and *Brassica rapa*. Multiple sequence alignment of GH1 proteins was performed using ClustalX2 with default parameters. The unrooted phylogenetic tree was constructed by MEGA 6 with the neighbor-joining (NJ) methods using the following parameters: bootstrap values (1,000 replicates) and Poisson model. The tree is divided into 11 phylogenetic subgroups, designated as GH1-a to GH1-k. Members of *Arabidopsis* and *B. rapa* are denoted by blue squares and red circles.

To understand the evolutionary relationship of the *BrBGLU* genes, phylogenetic analysis of the *BrBGLU* and *AtBGULU* genes was conducted. To obtain *AtBGLU*s, HMM searching was performed by using all of the putative protein sequences of the *Arabidopsis* genome (ARAPORT11, https://www.araport.org) as queries. A total of 48 *AtBGLU* genes were obtained, which agrees with the results of a previous study [6]. The 64 BrBGLUs and 48 AtBGLUs protein sequences were aligned using ClustaX2 [31]. An unrooted phylogenetic tree was constructed for the 64 BrBGLUs and 48 AtBGLUs, using the NJ method in MEGA6 with a Poisson model. All BGLU proteins were classified into 10 distinct subgroups, namely, BGLU-a to BGLU-j (Figure 2). The results of the phylogenetic analysis were relatively similar to the findings of a previous study using *Arabidopsis* [6], with a few exceptions. All *B. rapa* and *Arabidopsis* proteins are grouped into 10 subgroups, whereas *Arabidopsis* subgroups 8 and 9 were combined into a subgroup GH1-c in our analysis. In addition, AtBGLU48 (SENSITIVE TO FREEZING 2, SFR2), which belongs to a distinct lineage from 10 subgroups in a previous study [6,32], was incorporated into the GH1-j subgroup, together with BrBGLU8 and BrBGLU42 (Figure 2).

Phylogenetic analysis generated an interesting finding, that the clustering or groupings of genes were related to the chromosomal locus or function. Based on the functions of the *AtBGLUs*, flavonol accumulation (*AtBGLU1*-*6*) and anthocyanin glucosyltransferase (*AtBGLU7*-*11*)-related genes were highlighted by subgroup GH1-a [10,11,12]; flavonoid utilization-related *AtBGLUs* (*AtBGLU12*-*17*) were represented by the GH1-e subgroup [10,13]; myrosinase-encoding *AtBGLUs* (*AtBGLU34*-*39*) belonged to the GH1-d subgroup [14,15,16], and scopolin hydrolysis-related *AtBGLUs* (*AtBGLU21*-23) were grouped into GH1-i [21,22]. Most of the genes within the same clusters on a chromosome were grouped into the same subfamily, which is similar to the findings using *Arabidopsis*, i.e., *BrBGLU5*/*6*, *BrBGLU11*/*12*, *BrBGLU31*/*32*/*33*, *BrBGLU40*/*41*, *BrBGLU58*/*59*, and *BrBGLU61*/*62*. This clustering indicates that the *BGLU* genes may have evolved from an ancestral gene via gene duplication. However, *BrBGLU51* was grouped with six *AtBGLUs* (*AtBGLU34*/*35*/*36*/*37*/*38*/*39*) in the GH1-d subgroup, indicating the possible loss of some *BGLU* genes in *B. rapa*.

**Figure 3:**
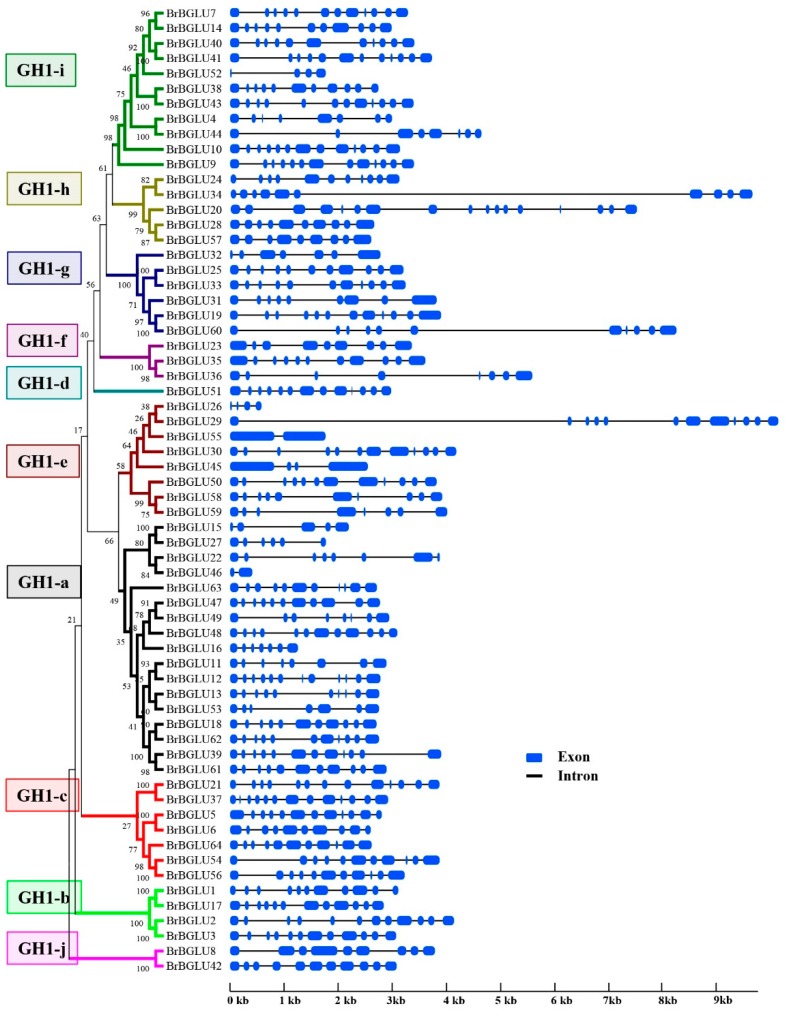
Exon–intron organization of *BrBGLUs* in different subgroups. Exons and introns are represented by blue boxes and black lines, respectively. The phylogenetic tree of each subfamily was constructed using MEGA6, as described in Figure 1.

Gene structure was commonly diversified during the evolution of the large number of gene families. To expand our knowledge of BrBGLUs in relation to evolution and functional diversification, the gene structures of the *BrBGLU*s were analyzed on the basis of exon–intron organization, using GSDS 2.0 [33]. The *BrBGLU*s exhibited 12 distinct exon–intron organization patterns, and the most common organization was 11 exons separated by 10 introns, presenting 19 members (Table 1 and Figure 3). Most genes contained more than two introns, except for *BrBGLU46* and *BrBGLU55*, indicating the possible occurrence of alternative splicing during gene expression. The *AtBGLU*s exhibited 10 distinct exon–intron organization patterns, and the pattern with 13 exons was the most common [6]. This analysis is consistent with *Arabidopsis* and rice, where the intron size and number of the *BGLUs* genes are highly variable [5,6].

### 2.3. Identification of BrBGLU Genes Involved in Pollen Development

Rice *TDR* (*Tapetum Degeneration Retardation*) mutant alters *BGLU1* expression with flower specificity [28], and *BGLU1* and *BGLU13* are found to be related to male organ development in *Calamus palustris* [34]. These previous reports lead to a hypothesis that *BrBGLU*s are involved in pollen development. To test this hypothesis, the previously published microarray data relating to male sterility in *B. rapa* [29] were re-annotated, using the improved *B. rapa* genome (version 3.0) [35] and analyzed based on pollen development (Table S1). A total of 36 *BrBGLU*s, represented by 88 probes (or 88 ESTs) showed significant hybridization values, of which 12 *BrBGLU*s showed over two-fold change in expression levels between fertile and sterile floral buds: six members were upregulated, and members were downregulated in fertile buds. Among these genes, four upregulated genes (*BrBGLU10/AtBGLU20, BrBGLU15/AtBGLU3, BrBGLU16/AtBGLU4*, and *BrBGLU64/AtBGLU41*) and two downregulated genes (*BrBGLU2/AtBGLU46* and *BrBGLU19/AtBGLU30*) were described as good candidates that were associated with pollen development. The function of all four upregulated genes has not been known up to now, but at least three, *BrBGLU10*, *BrBGLU15*, and *BrBGLU64* appeared to be related to pollen wall development. In particular, we further analyzed *BrBGLU10/AtBGLU20*, as these showed hundred-fold changes between fertile and sterile buds.

### 2.4. Analysis of the Putative Functions of BrBGLU10/AtBGLU20 in Pollen Development

**Figure 4:**
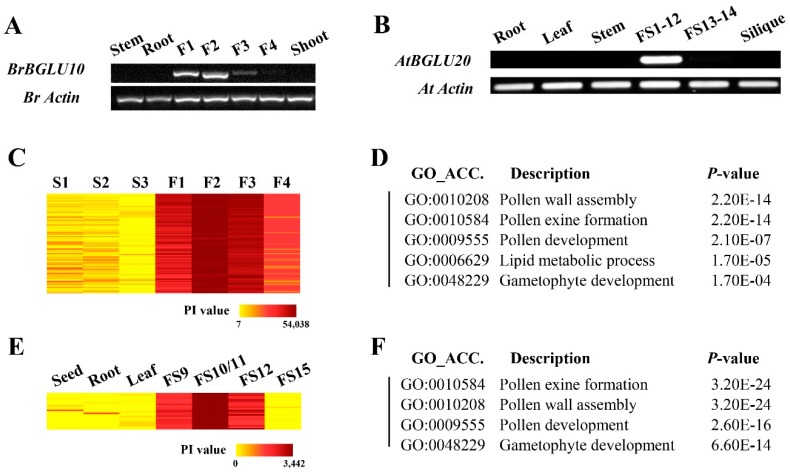
Analysis of expression of *BrBGLU10* and *AtBGLU20,* and Gene Ontology (GO) enrichment of co-expressed genes. **A**, Expression of BrBGLU10 in different tissues and floral bud stages in *B. rapa*. **B**, Expression of AtBGLU20 in different tissues and floral bud stages in *Arabidopsis*. **C**, Expression patterns of *BrBGLU10* and its co-expressed genes in sterile and fertile *B. rapa* floral buds, based on previously published microarray data [29]. **D**, GO enrichment analysis of genes co-expressed with *BrBGLU10*. **E**, Expression pattern of *AtBGLU20* and its co-expressed genes in various tissues of *Arabidopsis*, which was performed using the Arabidopsis eFP Browser (http://bar.utoronto.ca/efp/cgi-bin/efpWeb.cgi). **F**, GO enrichment analysis of genes co-expressed with *AtBGLU20.* S1–S3 represent the floral buds from male-sterile *B. rapa*. S1, before the tetrad stage. S2, after the tetrad stage. S3, containing aberrant pollen grains. F1–F4 indicate fertile *B. rapa* floral buds before the tetrad stage (F1), at the tetrad stage (F2), after the tetrad stage, but before containing mature pollen (F3), and containing mature pollen (F4). For *Arabidopsis*, FS1–12, flower stage 1 to stage 12; FS13–14, flower stage 13 to stage 14. PI, probe intensity.

To gain more insights into the functions of the *BrBGLU*s during pollen development, *BrBGLU10,* which was highly and specifically expressed in fertile buds, was selected for further analysis. *AtBGLU20,* the *Arabidopsis* ortholog of *BrBGLU10,* was initially named as *ATA27,* which is one of the *A. thaliana* anther-specific expressed genes [36]. To confirm the expression patterns of *BrBGLU10* and *AtBGLU20,* RT-PCR was conducted (Figure 4A,B). The expression level of *BrBGLU10* was specifically detected at the F1–F3 stages, with highest levels at the F2 stage, representing the tetrad stage, and *AtBGLU20* was specifically expressed before floral stage 12. The RT-PCR results might imply its important role in pollen development.

To demonstrate similar or conserved functions between *BrBLU10* and *AtBGLU20*, we isolated the co-expressed genes of *BrBGLU10*, using microarray data [29] and *AtBGLU20* from the *Arabidopsis* eFP Browser (http://bar.utoronto.ca/efp/cgi-bin/efpWeb.cgi) [37]. With the Pearson’s correlation coefficient (PCC) value above 0.90, 183 probes (107 genes) and 25 genes were determined to be co-expressed with *BrBGLU10* and *AtBGLU20,* respectively (Figure 4C, E; Tables S2–S3). *BrBGLU10* and its co-expressers were upregulated at the fertile floral bud stage, and the highest expression level was detected at the F2 stage (Figure 4C), suggesting that *BrBGLU10* plays a role during pollen development, especially from the tetrad stage to that before the mature pollen stage. In *Arabidopsis*, flower and stamen development processes were divided into 14 stages and 12 stages, respectively [37,38,39]. *AtBGLU20* and its co-expressers were represented by a high probe intensity (PI) value at flower stages (FS) 9 to 12, indicating that *AtBGLU20* plays a role in *Arabidopsis* pollen development (Figure 4E). We also conducted Gene Ontology (GO) enrichment analysis to provide more information on the function of *BrBGLU10* and *AtBGLU20* (Figure 4D,F). The results showed that genes involved in pollen exine formation and pollen wall assembly were highly over-represented among genes co-expressed with *BrBGLU10* and *AtBGLU20.* Taken together, our analysis indicated that *BrBGLU10* and *AtBGLU20* may be required for pollen development in both *B. rapa* and *Arabidopsis*.

**Figure 5:**
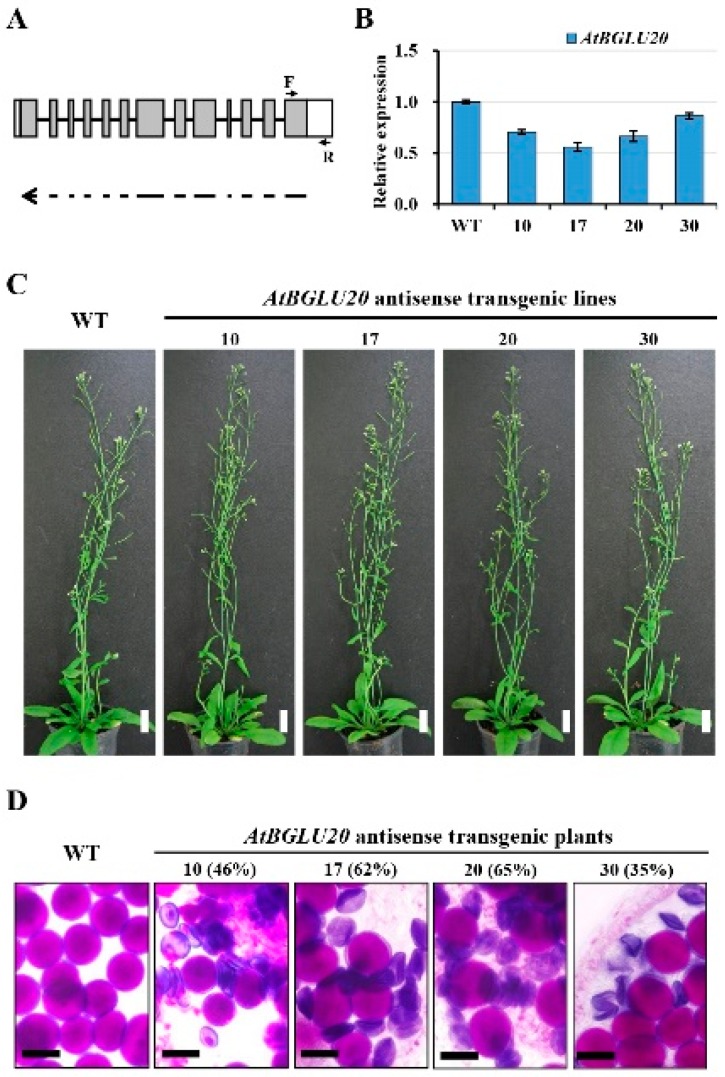
Analysis of WT and *AtBGLU20* antisense knockdown mutant *Arabidopsis* plants. **A**. Schematic representation of the *AtBGLU20* gene structure and DNA fragment regions for antisense constructs. The white box indicates the UTR region; gray boxes are exons; lines represent introns. The single arrow indicates the antisense orientation of the fragments in the constructs. F and R indicate the primer positions used in qRT-PCR analysis. **B**, Analysis of the expression levels of *AtBGLU20*. Expression was normalized to that of *At*ACT*7*, and represented relative to the expression levels of the WT. Error bars represent the SD of three biological replicates. **C,** Morphologies of wild-type Arabidopsis plants and *AtBGLU20* knockdown transgenic plants, which showed no obvious differences in vegetative growth. Bar = 20 mm. **D**, Mature pollen grain of WT and *AtBGLU20* transgenic plants stained with modified Alexander solution (Peterson et al., 2010). The well-developed pollen grains were stained red. Bar = 20 μm. WT, wild-type. 10, 17, 20, and 30 indicate four independent transgenic lines. The number in the parentheses indicate the percentages of defective pollen grains.

To validate *AtBGLU20* function in the pollen development, we generated knockdown mutants of *AtBGLU20* by introducing antisense constructs under the control of the *CaMV35S* promoter (Figure 5A). After screening, four independent knockdown lines were obtained with expression levels ranging from 55% to 85% (Figure 5B). However, the *AtBGLU20* downregulated plants showed normal vegetative growth based on morphology (Figure 5C), but produced defective pollen grains relative to the wild-type plants (Figure 5D). These results indicated that normal pollen development in *Arabidopsis* requires sufficient amounts of *AtBGLU20*. All data obtained from gene expression, co-expression analysis, and transgenesis led to the conclusion that *AtBGLU20* and *BrBGLU10* may have indispensable functions in pollen development in both *Arabidopsis* and *B. rapa*, respectively.

## 3. Discussion

### 3.1. Identification and Analysis of BrBGLUs

GH1 family genes play an important role in regulating abiotic and biotic stress responses, as well as various developmental processes in plants [9,12,14,18,23,40]. Based on the results of an increase in the number of whole genome sequences from a large number of species, genome-wide analysis of various gene families has been extensively performed. However, genome-wide identification and characterization of the GH1 gene family has only been reported in a few plant species, and there is no information on *Brassica* species, which are important crops for production of functional foods, as well as health-promoting compounds. In this study, the isolation of *BrBGLU*s from *B. rapa* genome (Figure 1), the distribution of *BrBGLU* genes on chromosomes (Figure 1), phylogenetic analysis (Figure 2), and exon–intron structures (Figure 3) provides substantial information on the functions and roles of these genes. 

Compared with the 49 *AtBGLUs* and 37 *OsBGLUs* in Arabidopsis and Rice, respectively [9], 64 *BrBGLUs* were isolated from the *B. rapa* genome, which is the largest number so far that has been reported in plants (Figure 1). The high number of *BGLU* family members in *B. rapa* could be related to the genome triplication event in this lineage [41]. To adapt different new functions that are suitable for changes in the environment, gene structure was commonly diversified during the evolution of multigene families [42]. For *BGLUs*, 13 exon–12 intron organization was considered as the ancestral gene structure, with the loss of certain introns leading to other gene structures [6]. The exons present in *BrBGLUs* varied from 2 to 13, and the most common organization was 11 exons (Figure 3). The introns in Arabidopsis vary from 0 to 13 [6]. This results suggested that little diversity exists in the gene structure of *BrBGLUs* when compared to *AtBGLUs*.

*BrBGLU*s may have originated from *Arabidopsis*, although duplication, gene loss, and functional diversification may have also occurred. This is supported by the fact that *BGLU*s from both species could be grouped into 10 subfamilies, with tandem arrays, as defined by Singh et al., 2013 [39], although some families were re-grouped or diverged into other subgroups. Figure 2 shows that *AtBGLU* subfamilies 8 and 9 [6] were incorporated into one *B. rapa* subfamily, GH1-c, and *BrBGLU51* is composed of GH1-d with six *AtBGLUs* (*AtBGLU34*/*35*/*36*/*37*/*38*/*39*), indicating the loss of some *BGLU*s in *B. rapa.* This phenomenon may result from the rapid evolution of genes similar to that previously observed between *Arabidopsis* and rice [5]. One more interesting finding was that *AtBGLU48* (*SFR2*) was incorporated into the GH1-j subgroup, with *BrBGLU8* and *BrBGLU42* (Figure 2). *AtBGLU42* is a β-glucosidase, but it is divergent from all other *AtBGLUs*, and more similar to several β-glycosidases from thermophilic archea and bacteria [32]. *SFR2* is involved in the lipid remodeling of the outer chloroplast membrane during freezing tolerance [43,44]. Because two *BrBGLU*s in the GH1-j subgroup had identities between 85% and 87% with *AtBGLU2*, *Brassica* genes may have a similar function of freezing tolerance as that in *AtSFR2*, although this requires further investigation.

On the basis of *Arabidopsis* study, most subfamilies of BGLUs in Figure 2 may be associated with specific functions: GH1-a for flavonoid and anthocyanin metabolism, GH1-e for flavonoid utilization, GH1-d for myrosinases, and GH1-i for scopolin hydrolysis. At least 12 genes are known to be involved in flavonoid metabolism in GH1-a: *AtBGLU1-6* for flavonol accumulation [10,11], *AtBGLU7-11* as anthocyanin glucosyltransferases [10,11,12], and *AtBGLU15* for flavonol bisglycoside catabolism under abiotic stress [13]. *AtBGLU12*-*17* in the GH1-e subgroup code for flavonoid-utilizing BGLUs in legumes [10]. An examination of the functions of *BrBGLUs* that are clustered with *AtBGLUs* in subgroups GH1a and GH1-e may provide information and understanding into the regulation of flavonoid biosynthesis in *Brassica* species.

Several subfamilies may be related to abiotic and biotic stress resistance, such as GH1-b, GH1-c, GH1-d, GH1-f, and GH1-i. Myrosinases hydrolyze glucosinolates into active forms that are involved in plant defense against herbivory and pathogens, and in human health promotion [45,46,47,48]. *AtBGLU26* and *AtBGLU34*-*39* function as myrosinases [14,15,16]. Except for *AtBGLU26* (GH1–h), most genes belong to the GH1-d subgroup (Figure 2). Understanding myrosinase function in Brassicaceae, which is rich in glucosinolates, may provide an excellent strategy for breeding health-promoting *Brassica* crops [49,50]. ABA also functions in stress responses, including drought stress. *AtBGLU18* [17] and *AtBGLU33* [18] hydrolyze glucose-conjugated ABA, thereby increasing ABA levels and inducing ABA responses such as drought tolerance. However, these two proteins are separated into two subfamilies, implying the presence of more BGLUs for the regulation of ABA levels. Scopolin is one of the coumarins produced in roots [51], and it plays a role in the defense against pathogen attack and abiotic stresses [19,20]. Three β-glucosidases that hydrolyze scopolin and their encoding genes (*AtBGLU21, 22* and *23*) have been characterized [21,22]. The GH1-i subfamily includes these three genes and 11 *BrBGLU*s, which should be examined in relation to scopolin production. The GH1-b subfamily includes two monolignol glucoside hydrolases (AtBGLU45 and AtBGLU46) that control lignin content [23]. Because *OsBGLU14*, *16*, and *18* are involved in lignin biosynthesis with monolignol β-glucosidase activity and compensate for the *Arabidopsis bglu45* mutant [52], BrBGLUs in this subfamily may play similar roles. *AtBGLU42* in GH1-c is involved in the induction of systemic resistance to bacterial disease, and the release of iron-mobilizing phenolic metabolites under iron deficiency [24]. Several genes in this subfamily would thus be expected to contribute to eliciting defense responses. All of this information may contribute to future research directions in relation to *BrBGLUs*.

### 3.2. The Potential Functions of BrBGLUs During Pollen Development

Previous studies on rice and other plant species have indicated that β-glucosidases play roles in pollen development [34,36,53]. To identify the BrBGLUs responsible for pollen development, the previously published microarray data relating to male sterility in *B. rapa* were re-annotated and re-analyzed. Among the 36 *BrBGLU*s, 12 *BrBGLUs* showed over a two-fold change between fertile and sterile floral buds (Table S1). However, six genes (four upregulated and two downregulated genes) were more extensively studied in terms of their role in pollen development. We selected one *BrBGLU10* for investigation, the homolog of *AtBGLU20*, which showed hundreds-fold changes in its expression.

We examined the expression levels of *BrBGLU10/AtBGLU20* and analyzed the co-expressed genes in both *B. rapa* and *Arabidopsis* (Figure 4). An assessment of expression levels strongly suggests that *BrBGLU10/AtBGLU20* are involved in pollen development. The cellular contents from the degeneration of the tapetum supports pollen wall formation and subsequent pollen release [39]. Mutations in polysaccharide metabolism-related genes lead to defective pollen wall formation [26]. Glycoside hydrolase has been reported to be involved in the cell wall polysaccharide degradation [27]. The expression patterns of *BrBGLU10* and *AtBGLU20* suggest that they might play a role from the tetrad stage to mature pollen grains (Figure 4A, B), which corresponding to the tapetum degradation stage [29,39]. Co-expression analysis is a valuable approach for classifying and visualizing transcriptomic data to identify genes with similar cellular functions and regulatory pathways [54,55,56], although this is not always the case [57,58]. In plants, co-expression analysis under various experimental conditions has been used for predicting gene function [55,59]. Figure 4C,D shows that this gene possibly regulates pollen development. In particular, GO annotation of co-expressed genes reflects that pollen wall and exine formation are influenced by *BrBGLU10/AtBGLU20*, indicating that the hydrolysis of glucose moieties is necessary for proper pollen development. 

Because BrBGLU10 had a high sequence identity with AtBGLU20 (87% at the nucleotide level and 84% at the amino acid sequence level), both genes may thus have similar functions. Therefore, knocking down *AtBGLU20* may provide direct evidence for its function in pollen development. Figure 5 shows that the suppression of *AtBGLU20* expression had no effect on plant growth and development, although this aborted pollen production. This result implies that BrBGLU10/AtBGLU20 are critical to pollen grain development.

## 4. Materials and Methods 

### 4.1. Plant Materials and Growth Conditions

Seeds of *B. rapa* subsp. *pekinensis* (Chiifu) were germinated in Petri dishes in the dark at 23 °C for two days, then the germinated seeds were transferred to a 4°C growth chamber with 16 h of light for 25 days to induce vernalization. After vernalization, the seedlings were transplanted into 15 cm × 15 cm × 18 cm pots containing potting soil and grown in a 23 °C growth chamber with 16 h of light. The floral buds were collected from 10 plants with three biological replicates, as previously described [29], and stored at −70°C until use. Root and shoot tissues were collected from three-week-old seedlings without vernalization. Stem tissue was sampled from the plants one week after bolting. 

*A. thaliana* (L.) Heynh var. Columbia (Col-0) plants were grown under 140 μmol/m^2^/s light intensity at 23 ± 1 °C with a long day cycle with 16 h of light for plant transformation. Seeds were sown in 55 mm × 55 mm pots in potting soil, stratified for three days at 4 °C, and then transferred to the growth room. The plants were then kept under a transparent polythene lid for one week to increase humidity and support equal germination. The plate-cultured seeds were sterilized with 30% bleach and 0.1% Triton X-100 (Sigma, St. Louis, MO, USA), stratified for three days at 4 °C, and sown in Petri dishes with dimensions of 100 mm × 100 mm × 20 mm. The dishes contained half-strength MS media (Duchefa Biochemie, Netherlands) supplemented with 0.8% phytoagar and 1% sucrose.

### 4.2. Antisense Constructs and Plant Transformation

The full-length coding sequence of *AtBGLU20* was cloned from first-strand complementary DNA (cDNA), using the primers BGLU20F (Table S4). Then, the fragments were inserted into T&A cloning vectors (RBC T&A cloning kit, Real Biotech Corporation, Taiwan). After confirmation of the *AtBGLU20* sequence in the T&A vector by sequencing, the fragment was cloned into pCambina 3300-35S binary vectors and used in plant transformation. Col-0 were used for transformation with *Agrobacterium tumefaciens* GV3101 carrying the above binary plasmid using the floral dip method [60]. The transformants were selected on plates containing 25 mg/mL glufosinate in MS medium (Sigma, St. Louis, MO, USA), and also confirmed by genomic DNA PCR analysis.

### 4.3. Reverse Transcription PCR and qRT-PCR

Total RNA (1 μg) from each sample was used in reverse transcription. First-strand cDNA was synthesized with a PrimeScript™ RT reagent kit with a gDNA Eraser kit (TaKaRa, Japan). The concentration of the synthesized cDNA was determined, and the cDNA was diluted to 20 ng/μL for PCR analysis. Semi-RT-PCR was performed, which consisted of denaturation at 94 °C 5 min; followed by 25 cycles of 94 °C for 30 s, 55 °C for 30 s, and 72 °C for 60 s. The qRT-PCR conditions were pre-denaturation at 95 °C for 30 s; followed by 30 cycles of 95 °C for 5 s, 60 °C for 20 s, and 72 °C for 15 s. All primer sequences used in this study are listed in Table S4. The semi-RT-PCR products were separated on 1.5% agarose gels, and stained with ethidium bromide. The qRT-PCR results were analyzed using the 2^−ΔΔC^_T_ method, with three biological replicates.

### 4.4. Pollen Viability

For pollen viability and pollen developmental progression, flowers collected from Col-0 and *AtBGLU20* antisense transgenic plants were fixed in Carnoy’s fixative (6:3:1 alcohol:chloroform:acetic acid) for 2 h. Then, the anthers were detected and stained with a solution of Malachite green, acid fuchsin, and Orange G for approximately 12 h, as previously described [61].

### 4.5. Identification of BrBGLUs and Phylogenetic Tree Construction

The protein sequence of 48 BGLU members were downloaded from TAIR (http://www.arabidopsis.org/tools/bulk/sequences/index.jsp) [6]. All putative protein sequences of *B. rapa* (version 3.0) were downloaded from BRAD (http://brassicadb.org/brad/index.php) [35] and used as queries to search against the Hidden Markov Model (HMM) profile (Version 3.1b2) with the Pfam HMM library (Pfam 32.0) [62]. A total of 64 protein sequences with PF00232 (E value below 1E^−5^) were obtained, and these sequences were considered as *BrBGLUs* candidates and used for further analysis. Multiple sequence alignment of full-length BGLU proteins and phylogenetic tree construction were conducted using ClustalX2 [31]. The phylogenetic tree was generated by the MEGA6 program, using the neighbor-joining method with the ‘pairwise deletion’ option and ‘Poisson correction’ model, with a bootstrap test of 1000 replications [63].

### 4.6. Chromosomal Location, Nomenclature, and Gene Duplication of BrBGLUs

The position of each *BrBGLU* on *B. rapa* chromosomes was identified from BRAD (http://brassicadb.org/brad/index.php). For nomenclature, the ‘*Br*’ for *B. rapa* was added, followed by BGLU, and numbered according to its position from top to bottom on *B. rapa* chromosomes 1–10. MCScanX software was used to search potentially duplicated BrBGLUs [64]. All of the putative protein sequences of *B. rapa* (version 3.0) were compared with themselves by BLASTP, with a tabular format and an e-value of < 10^−5^. Then, tandem, segmental, and dispersed duplications were identified using MCScanX, using default criteria.

### 4.7. Co-Expression and Gene Ontology Enrichment Analysis

*AtBGLU20* was used as bait gene for genome-wide co-expression analysis to identify genes of similar function from Expression Angler [65]. *BrBGLU10* was represented by two EST probes *Brapa_ESTC004210* and *Brapa_ESTC007739*, which were used as bait for co-expression analysis. A cutoff threshold of 0.90 for the Pearson correlation coefficient was used. The expression pattern analysis was performed using the Arabidopsis eFP browser (http://bar.utoronto.ca/efp/cgi-bin/efpWeb.cgi) [37]. Clustering analysis for categorization was performed with the TIGR Multi-Experiment Viewer (http://www.tm4.org/mev.html). GO enrichment analysis was performed using agriGO (http://bioinfo.cau.edu.cn/agriGO/index.php) [66].

### 4.8. Microarray Analysis

To analyze the gene expression patterns of *BrBGLUs* in *B. rapa* during pollen development, the previously published microarray data relating to male sterility analysis were downloaded from NCBI’s Gene Expression Omnibus (GSE47665) [29]. The microarray data were re-annotated using BLASTX by comparing with the newly improved *B. rapa* reference genome sequence (version 3.0) [35].

## 5. Conclusions

In conclusion, 64 *BrBGLUs* have been identified in *B. rapa* genome, which were classified into 10 subgroups with *Arabidopsis* counterparts, and the GH1-i subgroup included putative pollen development-related *BrBGLU10*. Base on its known function in *Arabidopsis*, BrBGLUs may participate in various defense responses against biotic and abiotic stresses, flavonoid metabolism, and pollen development. This study has provided valuable information for a better understanding of BGLUs, and for their biotechnological application to crops.

## Figures and Tables

**Table 1:** Characteristics of the GH1 (Glycoside hydrolase family 1) gene family in *Brassica rapa*.

Locus ID	Gene Name	CDS Length (bp)	Protein Length (aa)	Chromosome	Gene Start	Gene End	gDNA Length (bp)	No. of Exons	Best Hit to Arabidopsis (BLASTP)		
									ID	Gene Name	E-Value
*BraA01g012490.3C*	*BrBGLU1*	1290	430	Chr 01	6,516,500	6,519,450	2950	11	*AT4G21760*	*BGLU47*	0
*BraA01g029610.3C*	*BrBGLU2*	1452	484	Chr 01	19,673,693	19,677,620	3927	12	*AT1G61820*	*BGLU46*	0
*BraA01g029670.3C*	*BrBGLU3*	1551	517	Chr 01	19,772,218	19,775,132	2914	12	*AT1G61810*	*BGLU45*	0
*BraA01g032340.3C*	*BrBGLU4*	873	291	Chr 01	22,083,906	22,086,749	2843	8	*AT1G52400*	*BGLU18*	6.38 × 10^−85^
*BraA01g034680.3C*	*BrBGLU5*	1545	515	Chr 01	23,747,770	23,750,431	2661	12	*AT3G18080*	*BGLU44*	0
*BraA01g034690.3C*	*BrBGLU6*	1464	488	Chr 01	23,754,677	23,757,145	2468	10	*AT3G18070*	*BGLU43*	0
*BraA01g040820.3C*	*BrBGLU7*	1374	458	Chr 01	27,455,063	27,458,182	3119	12	*AT3G09260*	*BGLU23*	0
*BraA01g041990.3C*	*BrBGLU8*	1926	642	Chr 01	28,048,456	28,052,048	3592	9	*AT3G06510*	*BGLU48*	0
*BraA01g043570.3C*	*BrBGLU9*	1566	522	Chr 01	28,909,991	28,913,218	3227	13	*AT3G03640*	*BGLU25*	0
*BraA02g023150.3C*	*BrBGLU10*	1653	551	Chr 02	13,570,012	13,572,993	2981	13	*AT1G75940*	*BGLU20*	0
*BraA03g011770.3C*	*BrBGLU11*	894	298	Chr 03	5,059,601	5,062,343	2742	8	*AT1G45191*	*BGLU1*	2.53 × 10^−60^
*BraA03g011780.3C*	*BrBGLU12*	1023	341	Chr 03	5,063,808	5,066,446	2638	12	*AT1G60090*	*BGLU4*	9.15 × 10^−82^
*BraA03g024570.3C*	*BrBGLU13*	846	282	Chr 03	12,073,161	12,075,780	2619	10	*AT4G22100*	*BGLU3*	5.01 × 10^−68^
*BraA03g033950.3C*	*BrBGLU14*	1398	466	Chr 03	16,798,347	16,801,182	2835	11	*AT3G09260*	*BGLU23*	0
*BraA03g041420.3C*	*BrBGLU15*	729	243	Chr 03	20,778,980	20,781,062	2082	5	*AT4G22100*	*BGLU3*	2.54 × 10^−61^
*BraA03g041430.3C*	*BrBGLU16*	669	223	Chr 03	20,781,085	20,782,278	1193	7	*AT1G60090*	*BGLU4*	3.4 × 10^−100^
*BraA03g049730.3C*	*BrBGLU17*	1563	521	Chr 03	25,428,252	25,430,947	2695	12	*AT4G21760*	*BGLU47*	0
*BraA04g000610.3C*	*BrBGLU18*	1431	477	Chr 04	408,734	411,303	2569	11	*AT4G27830*	*BGLU10*	0
*BraA04g002030.3C*	*BrBGLU19*	1497	499	Chr 04	1,226,615	1,230,317	3702	12	*AT3G60140*	*BGLU30*	0
*BraA04g002040.3C*	*BrBGLU20*	2058	686	Chr 04	1,238,401	1,245,729	7328	18	*AT3G60120*	*BGLU27*	2.2 × 10^−149^
*BraA04g010020.3C*	*BrBGLU21*	1341	447	Chr 04	7,880,007	7,883,680	3673	13	*AT5G36890*	*BGLU42*	0
*BraA04g020960.3C*	*BrBGLU22*	891	297	Chr 04	15,776,965	15,780,643	3678	8	*AT5G44640*	*BGLU13*	1.5 × 10^−105^
*BraA04g023640.3C*	*BrBGLU23*	1638	546	Chr 04	17,341,351	17,344,539	3188	9	*AT2G32860*	*BGLU33*	0
*BraA04g031090.3C*	*BrBGLU24*	1233	411	Chr 04	21,218,491	21,221,708	3217	12	*AT3G60120*	*BGLU27*	0
*BraA04g031100.3C*	*BrBGLU25*	1380	460	Chr 04	21,229,282	21,232,323	3041	11	*AT5G24550*	*BGLU32*	0
*BraA04g031130.3C*	*BrBGLU26*	267	89	Chr 04	21,248,071	21,248,622	551	4	*AT2G44450*	*BGLU15*	5.66 × 10^−85^
*BraA04g031140.3C*	*BrBGLU27*	525	175	Chr 04	21,249,497	21,251,177	1680	6	*AT2G44450*	*BGLU15*	1.4 × 10^−110^
*BraA05g004330.3C*	*BrBGLU28*	1623	541	Chr 05	2,194,953	2,197,671	2718	11	*AT3G60120*	*BGLU27*	0
*BraA05g004340.3C*	*BrBGLU29*	1527	509	Chr 05	2,201,548	2,211,165	9617	12	*AT2G44450*	*BGLU15*	0
*BraA05g004350.3C*	*BrBGLU30*	1518	506	Chr 05	2,216,705	2,220,674	3969	12	*AT5G44640*	*BGLU13*	0
*BraA05g004360.3C*	*BrBGLU31*	1326	442	Chr 05	2,223,901	2,227,525	3624	9	*AT2G44460*	*BGLU28*	2.7 × 10^−131^
*BraA05g004370.3C*	*BrBGLU32*	1155	385	Chr 05	2,245,448	2,248,087	2639	7	*AT3G60140*	*BGLU30*	2.1 × 10^−140^
*BraA05g004380.3C*	*BrBGLU33*	1281	427	Chr 05	2,255,905	2,258,985	3080	11	*AT5G24540*	*BGLU31*	5.1 × 10^−145^
*BraA05g004390.3C*	*BrBGLU34*	1545	515	Chr 05	2,261,685	2,270,997	9312	11	*AT2G44490*	*BGLU26*	0
*BraA05g012860.3C*	*BrBGLU35*	1536	512	Chr 05	7,011,962	7,015,388	3426	11	*AT2G32860*	*BGLU33*	2.3 × 10^−162^
*BraA05g012870.3C*	*BrBGLU36*	957	319	Chr 05	7,023,185	7,028,485	5300	8	*AT2G32860*	*BGLU33*	4.3 × 10^−102^
*BraA05g015060.3C*	*BrBGLU37*	1461	487	Chr 05	8,601,511	8,604,284	2773	13	*AT5G36890*	*BGLU42*	0
*BraA05g017770.3C*	*BrBGLU38*	1278	426	Chr 05	10,758,114	10,760,718	2604	11	*AT1G52400*	*BGLU18*	0
*BraA05g033960.3C*	*BrBGLU39*	1434	478	Chr 05	24,329,347	24,333,054	3707	12	*AT4G27830*	*BGLU10*	0
*BraA05g037140.3C*	*BrBGLU40*	1332	444	Chr 05	25,685,745	25,688,976	3231	11	*AT3G09260*	*BGLU23*	0
*BraA05g037150.3C*	*BrBGLU41*	1374	458	Chr 05	25,691,345	25,694,889	3544	12	*AT3G09260*	*BGLU23*	0
*BraA05g038920.3C*	*BrBGLU42*	1782	594	Chr 05	26,547,600	26,550,524	2924	11	*AT3G06510*	*BGLU48*	0
*BraA06g002000.3C*	*BrBGLU43*	1347	449	Chr 06	1,220,707	1,223,925	3218	12	*AT1G52400*	*BGLU18*	1.5 × 10^−173^
*BraA06g011040.3C*	*BrBGLU44*	1080	360	Chr 06	5,995,931	6,000,341	4410	8	*AT3G21370*	*BGLU19*	0
*BraA06g024630.3C*	*BrBGLU45*	1599	533	Chr 06	17,098,530	17,100,946	2416	4	*AT5G44640*	*BGLU13*	0
*BraA06g038720.3C*	*BrBGLU46*	312	104	Chr 06	25,758,774	25,759,164	390	2	*AT4G22100*	*BGLU3*	8 × 10^−52^
*BraA07g008030.3C*	*BrBGLU47*	1434	478	Chr 07	8,145,282	8,147,911	2629	11	*AT1G60090*	*BGLU4*	0
*BraA07g008050.3C*	*BrBGLU48*	1428	476	Chr 07	8,161,734	8,164,666	2932	12	*AT4G22100*	*BGLU3*	0
*BraA07g011940.3C*	*BrBGLU49*	765	255	Chr 07	11,620,825	11,623,618	2793	8	*AT3G62750*	*BGLU8*	3.75 × 10^−29^
*BraA07g024150.3C*	*BrBGLU50*	1545	515	Chr 07	18,998,283	19,001,907	3624	12	*AT3G60130*	*BGLU16*	0
*BraA08g002600.3C*	*BrBGLU51*	1515	505	Chr 08	1,915,015	1,917,839	2824	13	*AT1G47600*	*BGLU34*	0
*BraA08g008860.3C*	*BrBGLU52*	408	136	Chr 08	7,848,512	7,850,189	1677	4	*AT3G09260*	*BGLU23*	1.03 × 10^−75^
*BraA08g014870.3C*	*BrBGLU53*	930	310	Chr 08	12,301,355	12,303,970	2615	7	*AT4G22100*	*BGLU3*	3.9 × 10^−127^
*BraA08g025770.3C*	*BrBGLU54*	1506	502	Chr 08	18,552,796	18,556,470	3674	11	*AT1G26560*	*BGLU40*	0
*BraA09g018020.3C*	*BrBGLU55*	1524	508	Chr 09	11,385,273	11,386,948	1675	2	*AT5G44640*	*BGLU13*	0
*BraA09g038410.3C*	*BrBGLU56*	1527	509	Chr 09	30,292,880	30,295,941	3061	11	*AT1G26560*	*BGLU40*	0
*BraA09g049950.3C*	*BrBGLU57*	1542	514	Chr 09	37,157,612	37,160,275	2663	10	*AT3G60120*	*BGLU27*	0
*BraA09g049960.3C*	*BrBGLU58*	1248	416	Chr 09	37,164,182	37,167,906	3724	10	*AT3G60130*	*BGLU16*	2.2 × 10^−165^
*BraA09g049970.3C*	*BrBGLU59*	1047	349	Chr 09	37,169,340	37,173,150	3810	8	*AT3G60130*	*BGLU16*	3.4 × 10^−165^
*BraA09g049980.3C*	*BrBGLU60*	1326	442	Chr 09	37,178,403	37,186,233	7830	11	*AT3G60140*	*BGLU30*	2.6 × 10^−179^
*BraA09g052040.3C*	*BrBGLU61*	1461	487	Chr 09	38,112,498	38,115,245	2747	11	*AT4G27830*	*BGLU10*	0
*BraA09g052050.3C*	*BrBGLU62*	1176	392	Chr 09	38,116,332	38,118,944	2612	11	*AT4G27830*	*BGLU10*	9.8 × 10^−140^
*BraA10g001490.3C*	*BrBGLU63*	1281	427	Chr 10	776,354	778,932	2578	11	*AT1G02850*	*BGLU11*	0
*BraA10g012660.3C*	*BrBGLU64*	1569	523	Chr 10	10,414,966	10,417,449	2483	11	*AT5G54570*	*BGLU41*	0

## Supplementary Materials

Supplementary materials can be found at https://www.mdpi.com/1422-0067/20/7/1663/s1.

## Abbreviations

GH1	Glycoside hydrolase family 1
BGLUs	β-glycosidase genes
BrBGLUs	*Brassica rapa* β-glycosidase genes
ABA	abscisic acid
OsTDR	Tapetum Degeneration Retardation
HMM	Hidden Markov Model
GO	Gene Ontology
CDS	coding sequence
